# *catena*-Poly[[tetra­aqua­copper(II)]-μ-tri­thionato-κ^2^ *O*:*O*′]

**DOI:** 10.1107/S1600536809047096

**Published:** 2009-11-14

**Authors:** Edward R. T. Tiekink

**Affiliations:** a Department of Chemistry, University of Malaya, 50603 Kuala Lumpur, Malaysia

## Abstract

The title supra­molecular polymer, [Cu(S_3_O_6_)(H_2_O)_4_]_*n*_, features a tetra­gonally distorted octa­hedral Cu^II^ centre within an O_6_ donor set with the longer Cu—O bonds linking the dication and the trithio­nate dianion. Extensive O—H⋯O hydrogen-bonding inter­actions connect the supra­molecular chains into a three-dimensional network.

## Related literature

For crystal structures containing the trithionate anion, see: Chun *et al.* (2000 ▶); Díaz de Vivar *et al.* (2005 ▶); Ferrari *et al.* (1977 ▶). For related copper(II) structures with bridging di-sulfonato ligands, see: Charbonnier *et al.* (1977*a* ▶,*b* ▶); Pasquale *et al.* (2007 ▶); Wang *et al.* (2005 ▶).
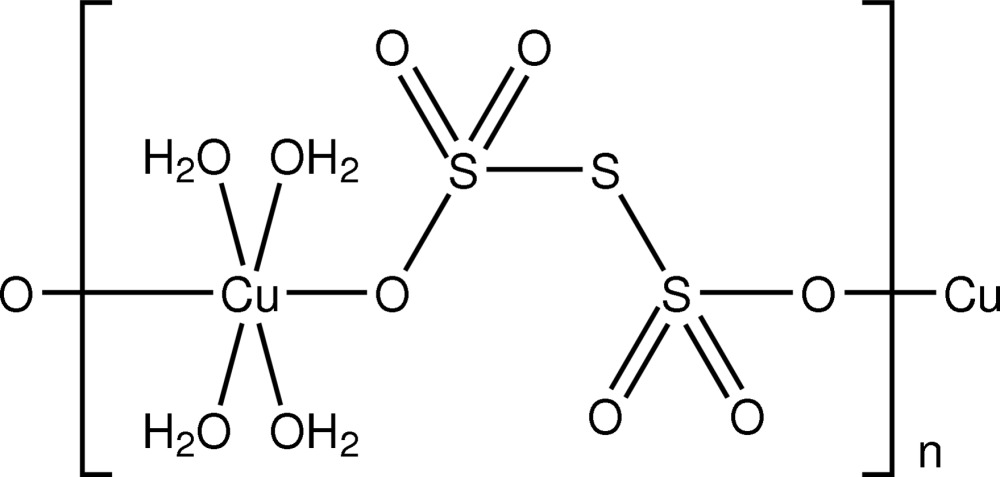


## Experimental

### 

#### Crystal data

[Cu(S_3_O_6_)(H_2_O)_4_]
*M* *_r_* = 327.78
Monoclinic, 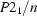

*a* = 7.1816 (1) Å
*b* = 21.4405 (4) Å
*c* = 7.7286 (1) Å
β = 116.092 (1)°
*V* = 1068.75 (3) Å^3^
*Z* = 4
Mo *K*α radiation
μ = 2.66 mm^−1^
*T* = 100 K
0.30 × 0.10 × 0.05 mm

#### Data collection

Bruker SMART APEXII diffractometer
Absorption correction: multi-scan (*SADABS*; Sheldrick, 1996 ▶) *T*_min_ = 0.911, *T*_max_ = 1
9081 measured reflections
2434 independent reflections
2291 reflections with *I* > 2σ(*I*)
*R*_int_ = 0.020

#### Refinement

*R*[*F* ^2^ > 2σ(*F* ^2^)] = 0.038
*wR*(*F* ^2^) = 0.108
*S* = 1.03
2434 reflections
151 parameters
12 restraints
H-atom parameters constrained
Δρ_max_ = 1.47 e Å^−3^
Δρ_min_ = −0.84 e Å^−3^

### 

Data collection: *APEX2* (Bruker, 2007 ▶); cell refinement: *SAINT* (Bruker, 2007 ▶); data reduction: *SAINT*; program(s) used to solve structure: *SHELXS86* (Sheldrick, 2008 ▶); program(s) used to refine structure: *SHELXL97* (Sheldrick, 2008 ▶); molecular graphics: *DIAMOND* (Brandenburg, 2006 ▶); software used to prepare material for publication: *SHELXL97*.

## Supplementary Material

Crystal structure: contains datablocks global, I. DOI: 10.1107/S1600536809047096/hb5217sup1.cif

Structure factors: contains datablocks I. DOI: 10.1107/S1600536809047096/hb5217Isup2.hkl

Additional supplementary materials:  crystallographic information; 3D view; checkCIF report

## Figures and Tables

**Table 1:** Selected bond lengths (Å)

Cu—O3	2.001 (3)
Cu—O1	2.002 (3)
Cu—O2	2.045 (3)
Cu—O4	2.047 (3)
Cu—O5	2.524 (3)
Cu—O8^i^	2.564 (3)

Symmetry code: (i) 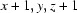.

**Table 2:** Hydrogen-bond geometry (Å, °)

*D*—H⋯*A*	*D*—H	H⋯*A*	*D*⋯*A*	*D*—H⋯*A*
O1—H1o⋯O9	0.84	2.29	3.080 (5)	157
O1—H2o⋯O9^ii^	0.84	2.25	3.071 (4)	166
O2—H3o⋯O10^i^	0.84	2.44	3.079 (5)	133
O2—H4o⋯O6^iii^	0.84	2.21	3.026 (4)	165
O3—H5o⋯O6^iii^	0.84	2.16	2.987 (4)	169
O3—H6o⋯O10^iv^	0.84	2.20	3.037 (5)	174
O4—H7o⋯O9^iv^	0.84	2.56	3.383 (5)	166
O4—H8o⋯O8^ii^	0.84	2.42	3.149 (4)	146

Symmetry codes: (i) 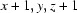; (ii) 
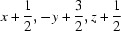
; (iii) 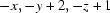; (iv) 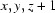.

**Fig. 1.**
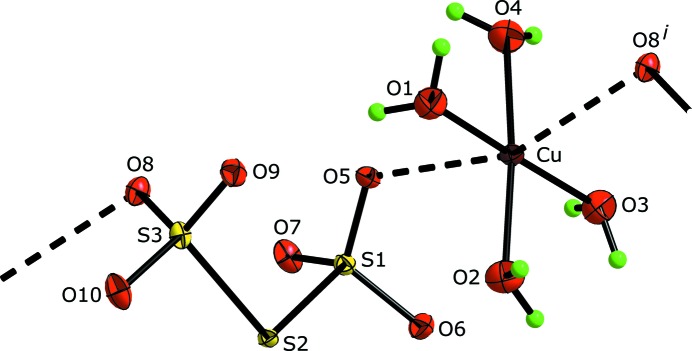
The asymmetric unit in (I) extended to show the coordination geometry about the Cu atom, showing displacement ellipsoids at the 50% probability level. Symmetry code: (i) 1 + x, y, 1 + z.

**Fig. 2.**
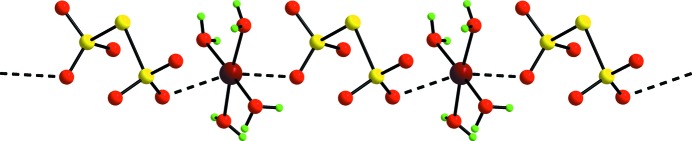
Supramolecular chain formation in (I). Colour code: Cu, brown; S, yellow; O, red; and H, green.

**Fig. 3.**
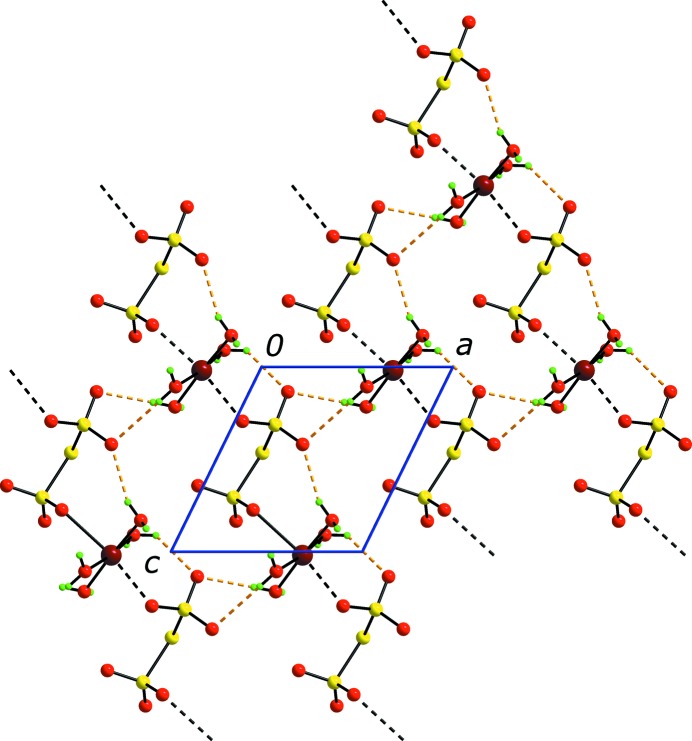
Supramolecular layer formation in (I) viewed in projection down the b axis. Chains shown in Fig. 2 are linked via O–H···O (orange dashed lines) hydrogen bonds. Colour code as for Fig. 2.

**Fig. 4.**
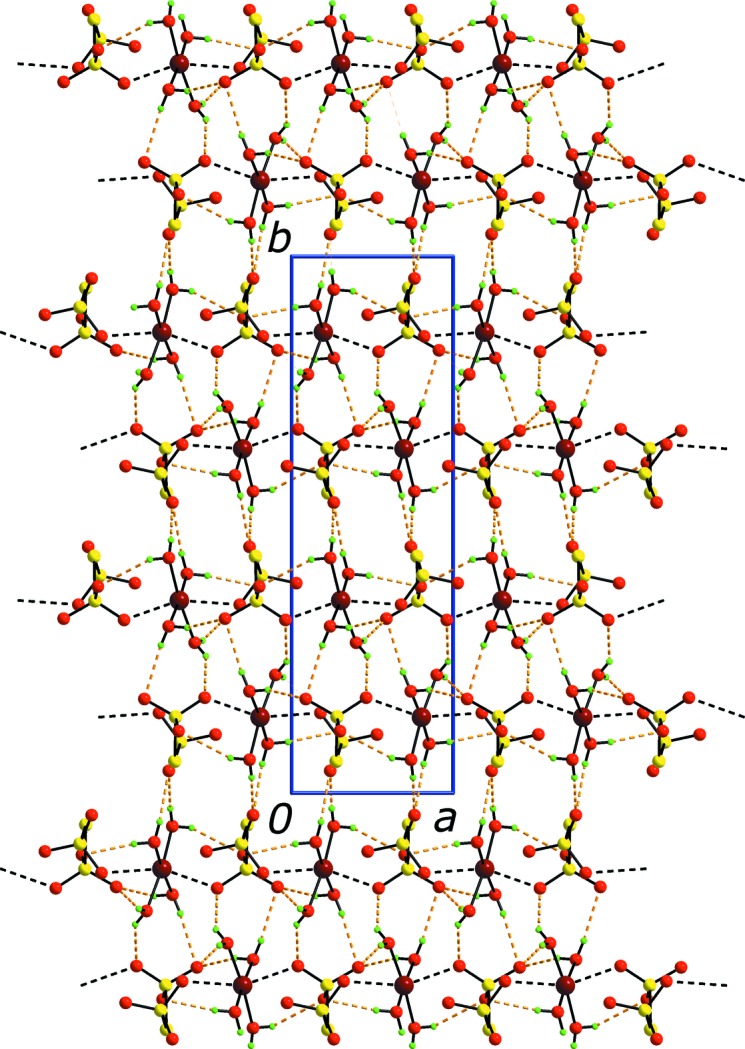
Unit-cell contents for (I) highlighting the 3-D network. The O–H···O hydrogen bonds are shown as orange dashed lines. Colour code as for Fig. 2.

## supplementary crystallographic information

### Comment

The title compound, (I), was obtained from an hydrothernal synthesis (see Experimental) and characterized crystallographically. The asymmetric unit comprises a tetraaqua copper(II) cation and a trithionato dianion, Fig. 1. In the crystal structure, the ions are connected as each trithionato dianion bridges two Cu^II^ centres thereby forming a supramolecular chain, Fig. 2. The Cu^II^ centre exists in a tetragonally distorted O_6_ octahedron with the Cu—O_aqua_ bonds [2.047 (3) - 2.047 (3) Å] being significantly shorter than the Cu—O_trithionato_ bonds [2.524 (3) and 2.564 (3) Å]. The Cu atom lies 0.025 (2) out of the basal plane defined by the four aqua-O atoms (RMS = 0.080 Å) in the direction of the O8^i^ atom, and the O5–Cu–O8^i^ axial angle is 157.74 (10) ° for i: 1 + *x*, *y*, 1 + *z*. Within the trithionato dianion, the S1–S2 and S2–S3 bond distances are 2.1450 (11) and 2.1184 (12) Å, respectively, and the S1–S2–S3 angle is 101.68 (5) Å. In the crystal structure, there is a considerable number of hydrogen bonding interactions. These occur within the supramolecular chain as well as between chains to form a 2-D array in the *ac* plane, Fig. 3. Connections between layers lead to a 3-D network, Fig. 4.

Structures containing the trithionato dianion are comparatively rare, with only three such examples (Chun *et al.*, 2000; Díaz de Vivar *et al.*, 2005; Ferrari *et al.*, 1977). In the same way, structures having [Cu(OH_2_)_4_]^2+^ centres bridged by bi-functional sulfonato ligands are uncommon (Charbonnier *et al.*, 1977*a*, b; Pasquale *et al.*, 2007; Wang *et al.*, 2005).

### Experimental

The blue crystal used in the present study was harvested from the hydrothermal reaction of stoichiometric amounts of CuCl, SeS_2_, S, and N_2_H_4_.H_2_O. The reagents were heated to 420 K for 3 d in a 25 ml Teflon-lined stainless-steel autoclave. After the reaction, the bomb was cooled to room temperature. The solution was filtered and layered with methanol. After four weeks, blue needles of (I) were collected and dried in air.

### Refinement

The O-bound H-atoms were located in a difference Fourier map and were refined with O–H and H···H restraints of 0.840±0.001 Å and 1.39±0.01 Å, respectively, and with *U*_iso_(H) = 1.5*U*_eq_(O).

### Crystal data

**Table:**

[Cu(S_3_O_6_)(H_2_O)_4_]	*F*(000) = 660
*M**_r_* = 327.78	*D*_x_ = 2.037 Mg m^−^^3^
Monoclinic, *P*2_1_/*n*	Mo *K*α radiation, λ = 0.71073 Å
Hall symbol: -P 2yn	Cell parameters from 6028 reflections
*a* = 7.1816 (1) Å	θ = 3.1–27.6°
*b* = 21.4405 (4) Å	µ = 2.66 mm^−^^1^
*c* = 7.7286 (1) Å	*T* = 100 K
β = 116.092 (1)°	Needle, blue
*V* = 1068.75 (3) Å^3^	0.30 × 0.10 × 0.05 mm
*Z* = 4	

### Data collection

**Table:**

Bruker SMART APEXII diffractometer	2434 independent reflections
Radiation source: sealed tube	2291 reflections with *I* > 2σ(*I*)
graphite	*R*_int_ = 0.020
φ and ω scans	θ_max_ = 27.5°, θ_min_ = 1.9°
Absorption correction: multi-scan (*SADABS*; Sheldrick, 1996)	*h* = −9→9
*T*_min_ = 0.911, *T*_max_ = 1	*k* = −27→27
9081 measured reflections	*l* = −9→10

### Refinement

**Table:**

Refinement on *F*^2^	Primary atom site location: structure-invariant direct methods
Least-squares matrix: full	Secondary atom site location: difference Fourier map
*R*[*F*^2^ > 2σ(*F*^2^)] = 0.038	Hydrogen site location: inferred from neighbouring sites
*wR*(*F*^2^) = 0.108	H-atom parameters constrained
*S* = 1.03	*w* = 1/[σ^2^(*F*_o_^2^) + (0.0493*P*)^2^ + 7.019*P*] where *P* = (*F*_o_^2^ + 2*F*_c_^2^)/3
2434 reflections	(Δ/σ)_max_ = 0.001
151 parameters	Δρ_max_ = 1.47 e Å^−^^3^
12 restraints	Δρ_min_ = −0.84 e Å^−^^3^

### Special details

**Table:**

Geometry. All s.u.'s (except the s.u. in the dihedral angle between two l.s. planes) are estimated using the full covariance matrix. The cell s.u.'s are taken into account individually in the estimation of s.u.'s in distances, angles and torsion angles; correlations between s.u.'s in cell parameters are only used when they are defined by crystal symmetry. An approximate (isotropic) treatment of cell s.u.'s is used for estimating s.u.'s involving l.s. planes.
Refinement. Refinement of *F*^2^ against ALL reflections. The weighted *R*-factor *wR* and goodness of fit *S* are based on *F*^2^, conventional *R*-factors *R* are based on *F*, with *F* set to zero for negative *F*^2^. The threshold expression of *F*^2^ > 2σ(*F*^2^) is used only for calculating *R*-factors(gt) *etc*. and is not relevant to the choice of reflections for refinement. *R*-factors based on *F*^2^ are statistically about twice as large as those based on *F*, and *R*- factors based on ALL data will be even larger.

### Fractional atomic coordinates and isotropic or equivalent isotropic displacement parameters (Å^2^)

**Table:**

	*x*	*y*	*z*	*U*_iso_*/*U*_eq_	
Cu	0.19601 (7)	0.85880 (2)	0.51918 (6)	0.01169 (14)	
O1	0.2615 (5)	0.81059 (15)	0.3309 (5)	0.0268 (7)	
H1O	0.1475	0.8079	0.2323	0.040*	
H2O	0.3104	0.7753	0.3752	0.040*	
O2	0.2609 (5)	0.93801 (15)	0.4087 (5)	0.0287 (7)	
H3O	0.3791	0.9353	0.4120	0.043*	
H4O	0.2480	0.9710	0.4611	0.043*	
O3	0.1488 (5)	0.90825 (15)	0.7159 (5)	0.0264 (7)	
H5O	0.1820	0.9459	0.7188	0.040*	
H6O	0.0233	0.9046	0.6919	0.040*	
O4	0.1007 (5)	0.77999 (16)	0.6068 (5)	0.0293 (7)	
H7O	0.0526	0.7840	0.6876	0.044*	
H8O	0.0311	0.7547	0.5190	0.044*	
S1	−0.31167 (13)	0.90509 (4)	0.20735 (12)	0.01136 (19)	
S2	−0.27573 (13)	0.94093 (4)	−0.03534 (11)	0.00925 (18)	
S3	−0.28093 (13)	0.85804 (4)	−0.18661 (12)	0.0127 (2)	
O5	−0.1785 (4)	0.85083 (12)	0.2738 (4)	0.0145 (5)	
O6	−0.2390 (5)	0.95873 (13)	0.3340 (4)	0.0194 (6)	
O7	−0.5286 (4)	0.89104 (14)	0.1427 (4)	0.0185 (6)	
O8	−0.4614 (4)	0.82243 (13)	−0.2060 (4)	0.0168 (5)	
O9	−0.0873 (4)	0.82490 (13)	−0.0809 (4)	0.0169 (5)	
O10	−0.2992 (4)	0.88484 (15)	−0.3653 (4)	0.0207 (6)	

### Atomic displacement parameters (Å^2^)

**Table:**

	*U*^11^	*U*^22^	*U*^33^	*U*^12^	*U*^13^	*U*^23^
Cu	0.0123 (2)	0.0112 (2)	0.0125 (2)	−0.00021 (15)	0.00630 (17)	0.00000 (15)
O1	0.0282 (16)	0.0247 (15)	0.0250 (16)	0.0013 (13)	0.0094 (13)	−0.0021 (12)
O2	0.0325 (17)	0.0244 (16)	0.0334 (18)	0.0006 (13)	0.0183 (15)	0.0002 (13)
O3	0.0279 (16)	0.0248 (15)	0.0277 (16)	−0.0018 (12)	0.0133 (14)	−0.0020 (12)
O4	0.0320 (17)	0.0271 (16)	0.0283 (17)	−0.0018 (13)	0.0130 (14)	0.0019 (13)
S1	0.0125 (4)	0.0115 (4)	0.0101 (4)	0.0007 (3)	0.0050 (3)	0.0010 (3)
S2	0.0132 (4)	0.0077 (4)	0.0085 (4)	−0.0006 (3)	0.0063 (3)	0.0003 (3)
S3	0.0111 (4)	0.0163 (4)	0.0106 (4)	0.0000 (3)	0.0047 (3)	−0.0002 (3)
O5	0.0164 (13)	0.0124 (12)	0.0133 (12)	0.0027 (10)	0.0052 (10)	0.0026 (9)
O6	0.0286 (15)	0.0139 (13)	0.0148 (13)	−0.0004 (11)	0.0087 (11)	−0.0020 (10)
O7	0.0131 (13)	0.0269 (15)	0.0162 (13)	0.0011 (11)	0.0071 (11)	0.0044 (11)
O8	0.0140 (12)	0.0183 (13)	0.0173 (13)	−0.0026 (10)	0.0063 (10)	−0.0024 (10)
O9	0.0137 (12)	0.0189 (13)	0.0164 (13)	0.0031 (10)	0.0049 (10)	−0.0007 (10)
O10	0.0183 (13)	0.0321 (16)	0.0130 (13)	0.0008 (12)	0.0081 (11)	0.0034 (11)

### Geometric parameters (Å, °)

**Table:**

Cu—O3	2.001 (3)	O3—H6O	0.8401
Cu—O1	2.002 (3)	O4—H7O	0.8401
Cu—O2	2.045 (3)	O4—H8O	0.8401
Cu—O4	2.047 (3)	S1—O7	1.443 (3)
Cu—O5	2.524 (3)	S1—O5	1.449 (3)
Cu—O8^i^	2.564 (3)	S1—O6	1.450 (3)
O1—H1O	0.8401	S1—S2	2.1450 (11)
O1—H2O	0.8401	S2—S3	2.1184 (12)
O2—H3O	0.8401	S3—O10	1.448 (3)
O2—H4O	0.8401	S3—O9	1.452 (3)
O3—H5O	0.8401	S3—O8	1.454 (3)
			
O3—Cu—O1	176.54 (14)	Cu—O4—H8O	116.6
O3—Cu—O2	91.32 (13)	H7O—O4—H8O	111.9
O1—Cu—O2	87.46 (13)	O7—S1—O5	113.51 (17)
O3—Cu—O4	89.60 (14)	O7—S1—O6	114.36 (18)
O1—Cu—O4	91.95 (14)	O5—S1—O6	114.32 (16)
O2—Cu—O4	174.05 (14)	O7—S1—S2	107.39 (12)
Cu—O1—H1O	104.4	O5—S1—S2	106.44 (12)
Cu—O1—H2O	111.0	O6—S1—S2	99.21 (12)
H1O—O1—H2O	111.9	S3—S2—S1	101.68 (5)
Cu—O2—H3O	110.1	O10—S3—O9	113.18 (17)
Cu—O2—H4O	113.9	O10—S3—O8	114.04 (17)
H3O—O2—H4O	111.5	O9—S3—O8	113.01 (17)
Cu—O3—H5O	112.9	O10—S3—S2	99.54 (13)
Cu—O3—H6O	107.9	O9—S3—S2	108.67 (12)
H5O—O3—H6O	111.2	O8—S3—S2	107.22 (12)
Cu—O4—H7O	118.0		
			
O7—S1—S2—S3	−78.83 (14)	S1—S2—S3—O10	168.90 (12)
O5—S1—S2—S3	43.05 (12)	S1—S2—S3—O9	−72.54 (13)
O6—S1—S2—S3	161.92 (13)	S1—S2—S3—O8	49.91 (13)

Symmetry codes: (i) *x*+1, *y*, *z*+1.

### Hydrogen-bond geometry (Å, °)

**Table:**

*D*—H···*A*	*D*—H	H···*A*	*D*···*A*	*D*—H···*A*
O1—H1o···O9	0.84	2.29	3.080 (5)	157
O1—H2o···O9^ii^	0.84	2.25	3.071 (4)	166
O2—H3o···O10^i^	0.84	2.44	3.079 (5)	133
O2—H4o···O6^iii^	0.84	2.21	3.026 (4)	165
O3—H5o···O6^iii^	0.84	2.16	2.987 (4)	169
O3—H6o···O10^iv^	0.84	2.20	3.037 (5)	174
O4—H7o···O9^iv^	0.84	2.56	3.383 (5)	166
O4—H8o···O8^ii^	0.84	2.42	3.149 (4)	146

Symmetry codes: (ii) *x*+1/2, −*y*+3/2, *z*+1/2; (i) *x*+1, *y*, *z*+1; (iii) −*x*, −*y*+2, −*z*+1; (iv) *x*, *y*, *z*+1.
